# The nonadaptive nature of the H1N1 2009 Swine Flu pandemic contrasts with the adaptive facilitation of transmission to a new host

**DOI:** 10.1186/1471-2148-11-6

**Published:** 2011-01-06

**Authors:** Juwaeriah Abdussamad, Stéphane Aris-Brosou

**Affiliations:** 1 Department of Biology, University of Ottawa, Ottawa, Canada; 2 Vellore Institute of Technology University, Vellore, India; 3 Center for Advanced Research in Environmental Genomics, University of Ottawa, Ottawa, Canada; 4 Department of Mathematics and Statistics, University of Ottawa, Ottawa, Canada

## Abstract

**Background:**

The emergence of the 2009 H1N1 Influenza pandemic followed a multiple reassortment event from viruses originally circulating in swines and humans, but the adaptive nature of this emergence is poorly understood.

**Results:**

Here we base our analysis on 1180 complete genomes of H1N1 viruses sampled in North America between 2000 and 2010 in swine and human hosts. We show that while transmission to a human host might require an adaptive phase in the HA and NA antigens, the emergence of the 2009 pandemic was essentially nonadaptive. A more detailed analysis of the NA protein shows that the 2009 pandemic sequence is characterized by novel epitopes and by a particular substitution in loop 150, which is responsible for a nonadaptive structural change tightly associated with the emergence of the pandemic.

**Conclusions:**

Because this substitution was not present in the 1918 H1N1 pandemic virus, we posit that the emergence of pandemics is due to epistatic interactions between sites distributed over different segments. Altogether, our results are consistent with population dynamics models that highlight the epistatic and nonadaptive rise of novel epitopes in viral populations, followed by their demise when the resulting virus is too virulent.

## Background

Viruses are the cause of several deadly diseases such as yellow fever, dengue, hepatitis or seasonal Influenza. The etiologic agent of the latter, the Influenza virus, can cause mild to severe illnesses depending on the Influenza type and strain. The case of the 2009 H1N1 outbreak, first detected in humans in early 2009 [1], was caused by a antigenically novel strain that led the World Health Organization to declare the outbreak as the first Influenza pandemic of the 21^st ^century. The emergence of such viruses in the human population has since attracted intense scrutiny, with a particular focus on two of their properties: virulence and interspecies transmission [2].

The H1N1 virus is an Influenza A virus that belongs to the family of orthomyxoviruses, and has a segmented negative single-stranded RNA genome made of eight segments that each encode 1-2 proteins necessary for virus attachment to host cells and spread of viral infection. By approximate order of decreasing sizes, these genes code for polymerase subunits (PB2, PB1 and PA), the hemagglutinin (HA) and neuraminidase (NA) antigens, a nucleoprotein (NP), a ribonucleoprotein exporter (NS2, also called NEP), an interferon antagonist (NS1), an ion channel protein (M2) and a matrix protein (M1). Two other proteins, PB2-F1 [3] and PB1-N40 [4], whose roles are now emerging, have also been characterized. This segmented genome is constantly evolving either by accumulating mutations, which generally lead to small antigenic differences ("antigenic drift") or by exchanging genomic segments, a process termed reassortment, which, when occurring between different subtypes, can lead to dramatic changes in antigenic properties, also called "antigenic shift" (e.g., [5]).

The actual changes that may have led to the emergence of past pandemics start to become clearer thanks to a number of studies. For instance, the first pandemic of the 20^th ^century in 1918, also known as 'Spanish Flu', was caused by an H1N1 virus, which was isolated and sequenced from a casualty preserved in the Alaskan permafrost [6]. Structural and genetic studies have shown that this particular 1918 virus lacked a cleavage site in HA [7], that virulence was determined by several proteins including HA, the replication complex, NS1 and PB1-F2 [2,8], while HA and PB2 played an important role in viral transmissibility [9]. The precise origin of this 1918 virus is however difficult to trace back in time due to the absence of genetic information on the viruses circulating before the 20^th ^century.

The emergence of the 2009 H1N1 pandemic is, on the other hand, not as well understood. Structural information revealed that the 2009 HA protein had a striking similarity to its 1918 counterpart [10]. In a landmark study, Smith and collaborators showed that the etiologic agent of the 2009 pandemic had three key features: (i) the polymerase genes as well as HA, NP and the NS genes emerged from triple reassortant North-American swine viruses while the NA and M genes originated from avian-like swine viruses, (ii) that the pandemic viruses diversified about a year before the onset of the pandemic and (iii) that a long branch separated the diversification of these pandemic viruses from their first emergence [11]. These authors suggested that the long branch leading to the diversification of the pandemic viruses both reflects a long unsampled history and mild evidence for positive selection, but they did not fully characterize the adaptive nature of the pandemic. It is also unclear whether the actual host-switch events from non-human animals to humans have an adaptive nature.

Here we revisit the adaptive nature of the 2009 H1N1 pandemic with a detailed analysis of the role of selection in (i) the emergence of this virus, and (ii) its adaptation to human hosts. On the basis of an extended data set compared to [11], we show that while the acquisition of efficient human-to-human transmission was driven by positive selection, the emergence of the 2009 H1N1 pandemic was essentially nonadaptive, and resulted from stochastic processes, which in turn are expected to make the prediction of such dramatic events difficult.

## Results and Discussion

### Phylodynamics of the 2009 H1N1 pandemic

We downloaded 1180 complete Influenza A genomes of the H1N1 subtype in North America collected between year 2000 and 2010, and selected only the gene sequences with at most 99.99% similarity for each of the ten "canonical" protein-coding genes (see Methods). This clustering step allowed us to performed all phylogenetic analyses in a reasonable timeframe while conserving most of the sequence diversity present in the original data set. In order to reconstruct rooted phylogenetic trees for each of these genes, we used the 'relaxed molecular clock' approach implemented in BEAST [12] (see [13] for rooting a tree with a clock), where tip dates were set to the collection year of each virus. A calibration scheme at a finer time-scale was not used because the information about the collection month was missing from some of the sampled genomes. The substitution models selected by the Akaike Information Criterion [14] were all GTR + Γ + I, except for PB1 (GTR + Γ), M2, M1 and NS2 (TVM + Γ) and NS1 (TVM + I). Since TVM-based models are not implemented in BEAUTi, we employed the next best AIC model which in all cases was based on GTR for the relaxed clock analyses with BEAST.

**Figure 1:**
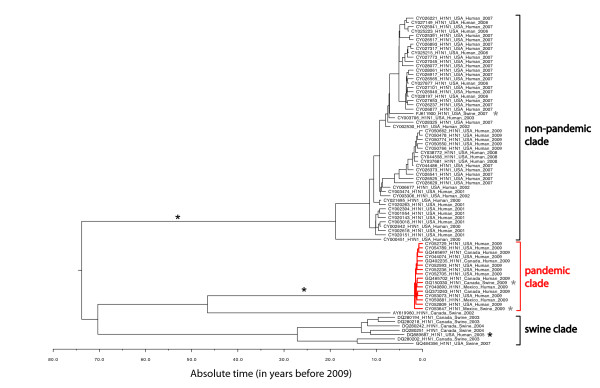
**Phylogeny estimated for the NA protein-coding gene**. Sequences from the 2009 H1N1 pandemics and the clade sustaining them are in red. Host switch events are marked with a star (*): in black for swine-to-human event and in gray for human-to-swine events. Branch lengths are proportional to time; the horizontal axis below the tree gives the scale.

The results show that H1N1 sequences across all ten genes have very similar histories (Figure 1 for the NA gene; see Additional File 1 and 2). If we assume that the ancestral H1N1 genome is of swine or other non-human origin [11], there were a minimum of three host-switch events to human: two occurred on internal ("deep") branches, one of which led to the 2009 pandemic. This particular host-switch event was placed on the long branch sustaining the 2009 clade rather than on the short branch leading to the Mexico_-_swine_-_2009 genome because the position of this latter genome within the 2009 clade is weakly supported (posterior probabilities ≤ 0.20 over the ten genes analyzed). The third host-switch event occurred on a terminal branch of the tree (Figure 1). It is notable that only one of the two internal host-switch events led to a pandemic, which suggests that the two processes of host-switch event and 'pandemicity' are not tightly coupled, as already suggested by the 2005 H5N1 viruses. In the rest of the text, we will denote the part of the tree that leads to the human 2009 pandemic sequences as the "pandemic clade", while all the other human sequences are part of the "non-pandemic clade" (Figure 1).

**Figure 2:**
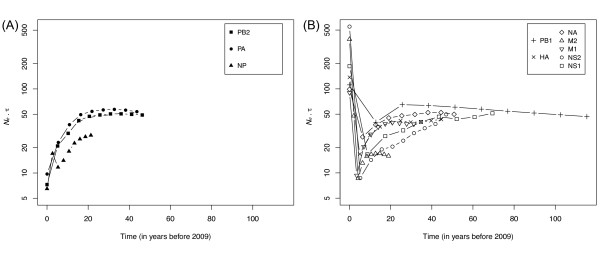
**Skyline reconstructions of the demographics of all ten protein-coding genes**. The effective population size (*N_e_*) scaled to generation time (*τ*) is plotted against absolute time, expressed in years before 2009. (A): for the three genes with decreasing *N_e _*× *τ *are represented by filled symbols. (B): for the seven genes with the recent increase in *N_e _*× *τ*.

The relaxed clock analyses also allow us to derive three additional results on (i) the population dynamics of the different segments and genes, (ii) their rates of evolution and (iii) their coalescence times. First, the results of the skyline analyses show that the population dynamics of the different segments and genes exhibit two very contrasted trajectories (Figure 2). While most segments followed similar and downwards dynamics in the past, a decoupling event or a series of such events took place at most five years before 2009, *ca*. 2004, when seven of the ten genes underwent a rapid expansion suggestive of a selective sweep. The time resolution of our analyses is too low for us to derive more accurate dates, but the suddenness of this expansion suggests that it would have been difficult to forecast as it represents a dramatic departure from the previously decreasing trend. These "expanding genes" include one of the polymerase genes (PB1), the two antigenic determinants (HA and NA), and the genes on the last two segments, M and NS. On the other hand, two of the polymerase genes (PB2 and PA) as well as the nucleoprotein (NP) underwent a steady decrease in terms of scaled effective population size (*N_e_τ *). Note first that these estimates are relative to the viral population, not to the host's dynamics, and therefore represent the *incidence *of the virus rather than its *prevalence *[15]. Second, segment dynamics are not linked to the origin of the segments or genes, as PA and NP, which come form a North American avian and a classical avian source, respectively [2], still exhibit similar dynamics (Figure 2A). Yet, such a decoupling of segment dynamics is not atypical in Influenza A viruses (see [16]). One notable difference with the latter study however is that our reconstruction goes 40 years back in time before the 2009 outbreak without encountering any of the oscillations reconstructed over a 14 year period for H3N2 viruses [16]. A potential explanation is that the pattern observed here is due to our smaller effective sample size (after clustering of sequences at the 0.01% similarity level), and/or to the lower temporal resolution of our analysis. In spite of these potential confounding factors, the lack of oscillations detected in our results might also reflect the lack of evidence for seasonality in H1N1 dynamics, which is consistent with the dominant incidence of H3N2 viruses in the human population between 1968 (the year of the 'Hong Kong Flu') and 2009 [16]. While in the face of the 2009 pandemic it makes sense that *N_e_τ *for both the HA and NA antigens increased, it is unclear (i) why *N_e_τ *decreased for some segments and (ii) why a decoupling is inferred within the polymerase genes, setting PB2, which has a role in host restriction (e.g., [17]), apart. This decoupling of segments cannot be due to our sequence clustering that eliminated highly similar sequences, but under-sampling of genomes cannot be ruled out (see below).

**Figure 3:**
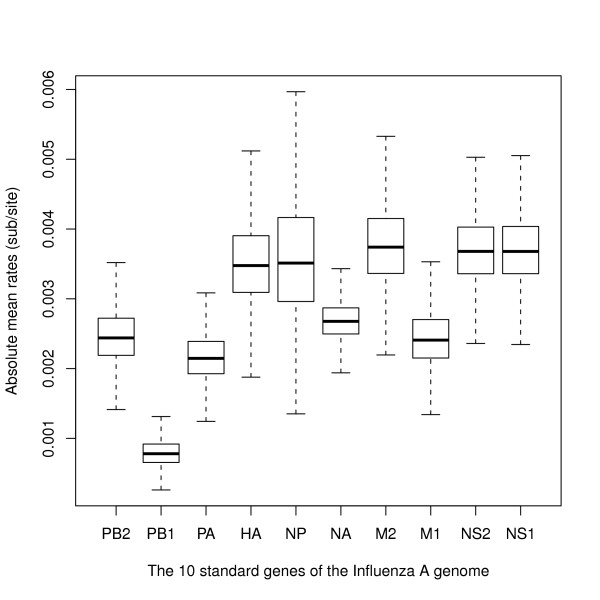
**Box-and-whisker plot of posterior absolute mean rates of evolution for all ten protein-coding genes**. The mean rates were sampled by BEAST from their respective posterior distribution.

Second, the posterior distributions of the absolute rates of evolution are summarized in Figure 3. These rates are similar to those estimated in previous studies (e.g., [11,16]), and our results suggest that there is extensive rate heterogeneity between the different segments of the Influenza A genomes of H1N1 viruses, and even within segments as demonstrated in particular by the posterior estimates for M2 and M1 (Figure 3). Post-hoc comparisons of rates sampled from their posterior distributions, either by means of Tukey HSD or pairwise *t *tests, show significant differences at the *α *= 0.001 level, even under the very conservative Bonferroni correction. Therefore, Influenza A viruses of different subtypes evolve at different rates as reviewed before [18], and each of their protein-coding genes, even on the same segment, exhibit significant rate heterogeneity. Summarizing rates of evolution of Influenza A viruses and possibly other segmented RNA viruses by a single number might therefore not give a realistic picture of the extensive rate variation found in these viruses.

Third, Table 1 shows that the pandemic and non-pandemic H1N1 protein-coding genes analyzed here coalesced on average 65 years before 2009, that is, around 1944 (SEM = 18.52 years, excluding PB1 and NS1). NS1 and in particular PB1 have both been circulating for much longer periods of time (since 1878 and 1728, respectively; Table 1). Keeping in mind that the accuracy of the estimated dates depends on the density of sampled genomes, three points can be made here: (i) these dates are much deeper than in [11], where time to the most recent common ancestor (TMRCA) of the sampled sequences goes back to ~ 1985, due to the lower breadth of their sampling strategy. The inclusion of the 1918 Brevig genome A/Brevig Mission/1/1918 [6] for instance would only pull this root age further back in time; (ii) we also detected variation of coalescence times within segments: the protein-coding genes on segment 7 and 8, M2-M1 on the one hand and NS2-NS1 on the other hand, coalesced at slightly different dates, although their 95% HPDs overlap - which might be due to a combination of short sequences and small numbers of variable sites in the overlapping genes on segments 7 and 8; (iii) the observation that different segments share the same coalescence times has already been documented and interpreted as evidence for the correlated evolution and co-transmission of segments [19], so that there would be genetic *linkage *between segments. However, [19] found that coalescence times were shared by the PB2/PB1/PA/NP/M segments, while our Table 1 suggests that PB2/PA/HA/NA/NS2 have similar root age and therefore could be linked. The different linkage groups or constellations might be a characteristic of the different viruses studied (avian Influenza viruses of different subtypes in [19]*vs*. H1N1 in human and swine here). However, it is also possible that such gene constellations are highly labile both in time and across subtypes.

**Table 1:** Estimated dates for the gene-specific ages of the root of the sampled H1N1 sequences, the divergence of the pandemic clade (MRCApandemic), and for the diversification of the 2009 pandemic sequences (Pandemic age).

	Root	95% HPD*_root_*	MRCA*_pandemic_*	${\text{HPD}}_{MRCA_{pandemic}}$	Pandemic age	95% HPD*_pandemic_*
PB2	74.28	105.75-46.09	8.54	12.06-5.35	1.00	1.60-0.48
PB1	281.40	491.80-109.34	15.44	23.53-9.30	4.37	6.65-2.32
PA	79.71	111.65-49.45	13.56	19.67-7.88	1.03	1.58-0.56
HA	83.87	129.77-45.50	7.79	12.57-3.96	1.19	1.62-0.82
NP	55.39	95.16-22.36	7.88	11.34-5.08	0.93	1.60-0.37
NA	74.61	93.17-57.07	43.03	56.95-28.56	1.53	2.08-0.94
M2	29.51	39.69-19.98	14.76	20.99-8.72	1.30	1.31-1.20
M1	48.69	66.69-31.12	34.07	49.57-17.94	1.70	2.63-0.88
NS2	70.70	97.57-46.96	7.35	9.67-5.38	1.22	1.84-0.81
NS1	130.95	189.46-78.78	9.16	12.32-6.38	1.11	1.55-0.76

All dates are in years before 2009.

Notes--HPD: Highest Posterior Density; MRCA: Most Recent Common Ancestor.

A way to test this lability hypothesis is to estimate the TMRCA of the pandemic sequences. Unlike the TMRCA of the sampled H1N1 sequences, the emergence of the pandemic sequences shows a very consistent date across all segments and genes at 1.22 years before 2009, that is during the last semester of 2007 (SEM = 0.25 year). This date is slightly older than previous estimates that put the TMRCA of pandemic sequences sometime between mid-2008 [11] to early January 2009 [1]; this difference can be due to relaxation of selective constraints that are not directly accounted for here [11], slight differences in model specifications ([1] used a coalescent prior with exponential growth rather than a skyline model used here) and to our generally broader (but less dense) sampling of genomes. Based on the synchrony argument used above and in [19], this result suggests the formation of a new gene constellation in the late 2007. The emergence of this constellation could be the consequence of a selective sweep, as suggested in the case of avian Influenza viruses [19], but it could also be due to a demographic bottleneck in the viral population or other nonadaptive processes.

The lability hypothesis has a corollary that is easily testable: although the reassortment events that led to the emergence of the pandemic strain have a history that goes back to the early 1990's [11], consistently with the TMRCA estimated here (MRCA*_pandemic _*in Table 1), the coalescence times of the genomes analyzed here occurred only shortly before the pandemic. The most recent common ancestor of the pandemic clade (MRCA*_pandemic_*) has a mean age of 16.16 years before 2009 (SEM = 12.36 years; Table 1), which corresponds to the end of 1992. The 14.94 years (= 16.16 - 1.22) gap separating this MRCA*_pandemic _*from the pandemic clade represents a long period of time when sequences leading to the 2009 pandemic were not sampled [11]. But the long branch leading to the pandemic clade (Additional File 2) could also be due to the simultaneous action of positive selection.

### Test of positive selection for the 2009 H1N1 pandemic

To test the hypothesis that the long branch leading to the 2009 pandemic might represent the action of positive selection, we performed a branch-site test of positive selection along this branch in all ten protein-coding genes of the H1N1 Influenza A genome. The results, presented in Table 2 demonstrate quite dramatically that none of the ten protein-coding genes shows any evidence for positive selection, hereby suggesting that this long branch reflects exclusively a period of 15 years of unsampled history, and hence a dramatic failure of the current surveillance system of circulating Influenza viruses [11].

**Table 2:** Neutrality tests and test of positive selection for the human 2009 H1N1 pandemic.

	Model	*p*-MKT	*p_D_*	*np*	ln *L*	*p*-value	*ω*	***p***_***ω***_	sites (95%)
PB2	*H*_0_			108	-10489.98		0.037	0.967	na
	*H*_1_	0.770	0.573	109	-10489.98	1.000	1.000	0.000	none
PB1	*H*_0_			116	-10151.99		0.023	0.940	na
	*H*_1_	0.671	0.072	117	-10151.99	1.000	0.023	0.940	none
PA	*H*_0_			110	-9736.96		0.032	0.955	na
	*H*_1_	0.327	0.169	111	-9736.96	0.999	0.032	0.955	none
HA	*H*_0_			158	-10294.19		0.067	0.862	na
	*H*_1_	0.844	0.026	159	-10293.78	0.362	1.000	0.007	none
NP	*H*_0_			110	-6402.29		0.039	0.969	na
	*H*_1_	0.982	0.392	111	-6402.29	0.989	1.000	0.000	none
NA	*H*_0_			154	-8085.11		0.075	0.846	na
	*H*_1_	0.015	0.034	155	-8085.01	0.655	2.345	0.001	none
M2	*H*_0_			224	-1699.98		0.132	0.000	na
	*H*_1_	0.436	0.007	225	-1699.98	0.976	1.171	0.384	none
M1	*H*_0_			142	-3175.82		0.025	0.973	na
	*H*_1_	0.894	0.101	143	-3175.82	1.000	1.000	0.000	none
NS2	*H*_0_			286	-2341.50		0.082	0.865	na
	*H*_1_	0.901	0.005	287	-2341.50	0.984	1.000	0.000	none
NS1	*H*_0_			182	-4142.63		0.125	0.696	na
	*H*_1_	0.271	0.011	183	-4142.63	1.000	1.000	0.001	none

Results of the McDonald-Kreitman test (MKT; *p*-value), Tajima's *D *test (*p*_*D*_), as well as log-likelihood values (ln *L*), parameter estimates and *p*-values for the ten standard protein-coding genes of 2009 H1N1 Influenza A genomes. *H*_0 _is the null branch-site codon model, without positive selection; *H*_1 _is the alternative model that allows for positive selection at sites in the foreground branches. The *p*-values are derived from a $\chi_{1}^{2}$ distribution (see Methods). Parameter estimates for *ω *and its proportion of sites *p*_*ω *_are for rate categories *ω *< 1 under *H*_0_, and *ω *≥ 1 under *H*_1_.

Notes--na: not applicable; nfd: no fixed differences. Sites in boldface are those for which the LRT rejects *H*_0 _at the 1% level.

One potential caveat with our analysis is that codon models assume that all nonsynonymous differences observed in the data are fixed [20]. However, when data are sampled at the population level, as is most likely the case here, it is possible that most of the observed differences do in fact represent segregating polymorphisms. This is known to render the use of non-synonymous to synonymous rate ratios (*ω*'s) potentially problematic, as estimated *ω *ratios can take values < 1 within a population even in the presence of very strong positive selection [21]. As some of the nonsynonymous differences in our data are potentially transient polymorphisms, we reanalyzed the same data with two tests based on population genetics principles. First, we employed the McDonald-Kreitman test (MKT), which is a two-population neutrality test that compares the ratio of fixed nonsynonymous to synonymous differences to the ratio of polymorphic nonsynonymous to synonymous differences [22]. Here, a first "population" consisted of the sequences from the pandemic clade, while the other "population" contained all the remaining sequences in order to match the specification of the codon-based test of positive selection. The results suggest that there is no evidence for selection at the 1% level (Table 2), which supports the results of the likelihood ratio test based on codon models. Second, the results of the Tajima test, which compares two different estimates of nucleotide diversity under the infinite-site model, appear more contrasted, with only three genes (PB2, PA and NP) for which neutrality cannot be rejected. This set of genes matches exactly the list of genes with decreasing incidence (*N_e_τ*). Alternatively, the genes with an indication that neutrality could be rejected (PB1, HA, NA, the M and NS genes) are those that underwent a rapid and recent expansion. The results of the Tajima test are therefore potentially compounded by the effect of a recent "population" expansion of these segments, which is known to inflate the type-I error rate of this test [23]. In the absence of a clear rejection of the neutral hypothesis both with population genetics and phylogenetic approaches, the emergence of the 2009 H1N1 pandemic was therefore most likely due to nonadaptive processes such as drift (e.g., [24]).

### Test of positive selection for swine-to-human host-switch events

A complementary hypothesis is that the acquisition of the competence to be transmitted between humans requires some adaptive changes in the genome of the H1N1 virus of non-human origin. To evaluate this hypothesis, we first performed the MKT, defining the first "population" as that of viruses found in swines, and the second "population" as that of viruses found in humans. The results show that all the comparisons of nonsynonymous/synonymous polymorphisms to nonsynonymous/synonymous fixations failed because of the systematic absence of fixed differences (Table 3). This result raises some concern about saturation, which is probably not an issue here since the longest branch length is ≤ 0.2 substitutions per site, except for NS1 due to the presence in our data of a swine virus sampled in 2002 (which is actually an avian NS1-allele B; see Additional File 2). The results were identical when this sequence was excluded from the data. The absence of fixed differences, whose deficit usually indicates the action of purifying selection, here might also suggest that the MKT is not the most appropriate test for these data. This interpretation is supported by a simulation study that shows that the MKT exhibits unduly high type-I error rates at the large mutation rates (scaled to effective population sizes) typically found in RNA viruses such as Influenza viruses [23]. On the other hand, the Tajima test failed to reject the null hypothesis of neutral evolution for all protein-coding genes. Although all *p*-values are far from the 1% threshold used here (Table 3; except for HA, see below), this test has been shown to be conservative [25].

**Table 3:** Neutrality tests and test of positive selection for the H1N1 host-switch events.

	Model	*p*-MKT	*p_D_*	*np*	ln *L*	*p*-value	*ω*	***p***_***ω***_;	sites (95%)
PB2	*H*_0_			108	-10489.86		0.037	0.964	na
	*H*_1_	nfd	0.864	109	-10489.86	1.000	1.000	0.000	none
PB1	*H*_0_			116	-10150.60		0.022	0.960	na
	*H*_1_	nfd	0.528	117	-10150.60	1.000	0.022	0.960	none
PA	*H*_0_			110	-9736.96		0.032	0.955	na
	*H*_1_	nfd	0.940	111	-9736.96	1.000	1.000	0.000	none
HA	*H*_0_			158	-10294.27		0.067	0.862	na
	*H*_1_	nfd	0.066	159	-10288.97	0.001	2.190	0.003	**D144T; G172N**
NP	*H*_0_			110	-6402.15		0.037	0.948	na
	*H*_1_	nfd	0.965	111	-6402.15	1.000	1.000	0.001	none
NA	*H*_0_			154	-8083.65		0.075	0.728	na
	*H*_1_	nfd	0.611	155	-8078.72	0.002	8.469	0.002	**V80K; Q250A; F351Y**
M2	*H*_0_			224	-1700.43		0.136	0.611	na
	*H*_1_	nfd	0.824	225	-1697.33	0.013	34.479	0.010	none
M1	*H*_0_			142	-3173.37		0.021	0.941	na
	*H*_1_	nfd	0.569	143	-3173.37	1.000	1.000	0.001	(1 site)
NS2	*H*_0_			286	-2341.50		0.082	0.865	na
	*H*_1_	nfd	0.230	287	-2341.50	0.981	1.000	0.000	none
NS1	*H*_0_			182	-4142.63		0.125	0.699	na
	*H*_1_	nfd	0.334	183	-4142.63	1.000	1.000	0.000	none

Results of the McDonald-Kreitman test (MKT; *p*-value), Tajima's *D *test (*p*_*D*_), as well as log-likelihood values (ln *L*), parameter estimates and *p*-values for the ten standard protein-coding genes of 2009 H1N1 Influenza A genomes. *H*_0 _is the null branch-site codon model, without positive selection; *H*_1 _is the alternative model that allows for positive selection at sites in the foreground branches. The *p*-values are derived from a $\chi_{1}^{2}$ distribution (see Methods). Parameter estimates for *ω *and its proportion of sites *p*_*ω *_are for rate categories *ω *< 1 under *H*_0_, and *ω *≥ 1 under *H*_1_.

Notes--na: not applicable; nfd: no fixed differences. Sites in boldface are those for which the LRT rejects *H*_0 _at the 1% level.

From a phylogenetic standpoint, the test of positive selection based on codon models, although used conservatively here (see Methods), detected evidence for positive selection at the 1% level in the HA and the NA genes (Table 3). The HA gene codes for the principal surface antigen which is responsible for viral binding to host receptors via receptor-binding pockets, permitting entry into the host cell by membrane fusion and endocytosis [26]. As such, the HA gene appears to be a critical factor for efficient transmission from host to host. The NA gene codes for a tetrameric protein that facilitates the release and spread of viral particles to neighboring cells by cleaving sialic acids from infected cell surfaces and newly formed viral particles (e.g., [27]). Note that no evidence for adaptive evolution during host-switch events was found in the PB2 gene, which is often associated with host restriction (e.g., [17]). This lack of evidence for adaptive evolution during host switch might in turn be associated with the mild symptoms of the 2009 H1N1 pandemic viruses in the human population (e.g., [1]).

Of the two amino-acid sites in HA that were potentially under positive selection for adaptation to human hosts, only site 172 (158 in [10]) belongs directly to one of the four epitopes proximal to the receptor-binding pocket, while site 144 (131 in [10]) is two positions downstream of sites participating in the exact same epitope ('Sa' in [10]). It is therefore very likely that these two amino-acid changes improve viral binding to human hosts.

On the other hand, the interpretation of the results for NA is not so clear. Of the three amino-acid sites in NA that were identified to play a potential role in the adaptation to human hosts (Table 3), site 80 does not belong to the part of the NA protein that is usually crystalized (see [27]), and therefore its functional role is difficult to predict. Site 250 is in proximity of the catalytic pocket, but is not part of the sites that interact directly with the substrate (which are: 118, 151, 152, 224, 276, 292, 371, and 406; e.g. [27]). Site 351 is located in a loop that contributes to the binding of two antibodies [28]. It should be emphasized that the identification of sites under positive selection is difficult and not always absolutely reliable [29]. In spite of this, because our aligned sequences are quite conserved, it is unlikely that an unreliable alignment might have caused false positive identification of sites potentially under selection [30]. Therefore, we posit that the identified sites might play a role in reshaping the antigenic properties of the NA protein during the host-switch event from swine to human H1N1 viruses.

### Characterization of the pandemic N1 structure and epitopes

In order to test the hypothesis that the three sites identified above with the codon models do affect the antigenic properties of the NA protein, we first set out to predict the structural changes involved between pandemic and non-pandemic molecules.

**Figure 4:**
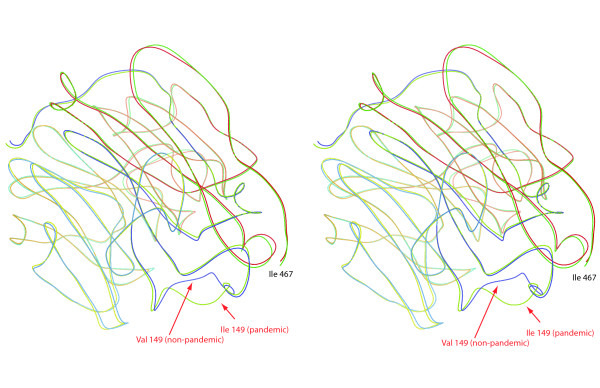
**Stereoscopic view of the aligned structural models of pandemic and non-pandemic NA proteins**. The major difference between these two partial models (covering residues 83-467) is highlighted and is due to one amino acid difference at position 149.

Recent work has focused on the HA protein, and has shown that the 1918 and 2009 proteins have very similar tridimensional (3D) structures [10]. Unfortunately, to date, the structure of the NA protein has not been obtained. We therefore reconstructed 3D models by homology modeling with Swiss-Model [31]. The prediction of the pandemic NA was based on PDB template 2HTY (Protein Data Bank identifier; E-value < 10^-500^), which is of type N1 but derived from an H5N1 virus isolated in 2004 [32], which is consistent with the avian origin of H1 [11]. Both the non-pandemic and the swine models were derived from PDB template 3B7E (E-value < 10^-500^), which is the structure of the pandemic 1918 N1 [27]. The three models covered residues 83-467 and were quite similar (RMSD*_ swine vs. nonpandemic _*= 0.15 Å; RMSD*_ pandemic vs. nonpandemic _*= 0.65 Å). The main structural difference between the pandemic and the non-pandemic structure is due to a nonsynonymous substitution in the 150 loop, which contains the active site of NA, at position 149 (Figure 4). Most of the pandemic sequences have an isoleucine at this position, just like the swine sequences isolated in 2009. On the other hand, all the non-pandemic sequences and swine sequences isolated before 2009 have a valine, which is also the amino acid used by the 1918 H1N1 NA protein and that was found to distort the structure of the NA protein [27]. This V149I substitution was previously reported in the context of studying two antineuraminidase compounds (zanamivir and oseltavimir), and was judged to be too far from the drug binding pocket to impact antiviral susceptibility [33]. Our results therefore suggest that (i) the V149I substitution affected the active site of NA but (ii) could not be solely responsible for the onset of the pandemic since the 1918 protein had a valine, unless epistatic interactions exist. Because we have found evidence for linkage between the segments of H1N1 (pandemic clade), such epistatic interactions cannot be ruled out; but since we found that all segments had the same TMRCA, our current approach cannot identify the segment(-s) and the site(-s) interacting directly with loop 150 of NA. However, in spite of this change in 3D structure of the pandemic NA protein, site 149 which is at the core of this structural change is not a site detected by our analyses of positive selection. Could nonetheless loop 150 still be a strong epitope?

**Figure 5:**
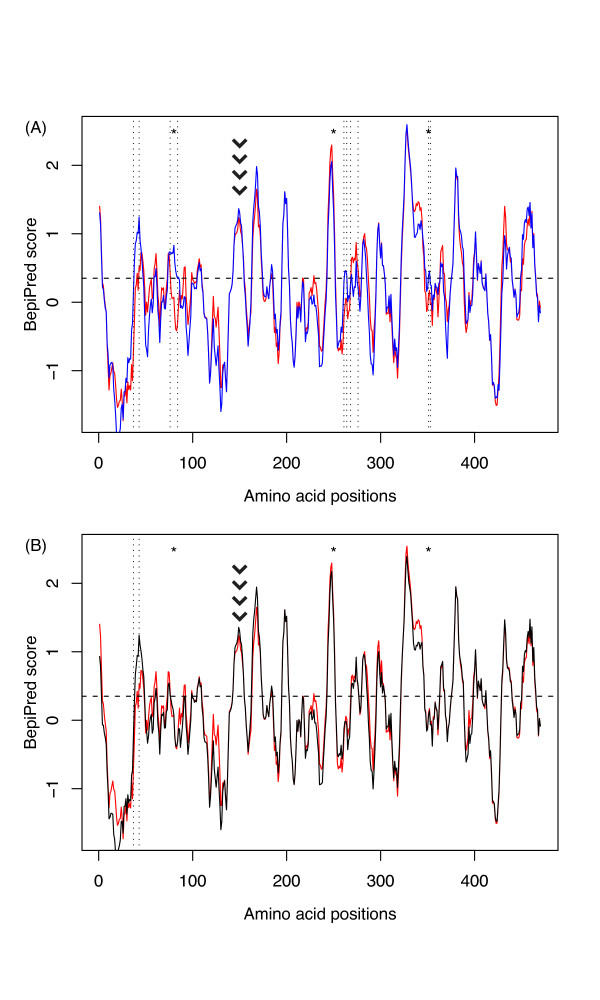
**Predicted B-epitopes of the NA protein**. (A) Comparison of pandemic human (red) *vs*. non-pandemic human (blue). (B) Comparison of pandemic human (red) *vs*. swine (black) predictions. The BepiPred score is represented as a function of the amino acid position along the protein. Scores above the 0.35 threshold (horizontal broken line) are considered significant (see Methods). Epitope differences between pandemic and non-pandemic NA proteins are highlighted for regions 37-43, 76-84, 261-264, 268-276, and 351-353 (dotted vertical lines). Asterisks (*) indicate the sites identified to be under positive selection during host-switch events. The chevron pattern locates loop 150.

To address this question, we predicted the epitopes of the non-pandemic NA proteins. Figure 5 shows the epitopes predicted for the human and swine sequences, with each peak above the 0.35 threshold indicating the presence of an epitope. More specifically, Figure 5A shows that a small number of differences exist between the pandemic and the non-pandemic proteins, as a total of five regions differ (37-43, 76-84, 261-264, 268-276, and 351-353). Notably, the sites detected to be potentially under positive selection for host-switch events, at positions 80, 250 and 351, are included or in very close proximity of the regions where epitope differences are detected between pandemic and non-pandemic NA proteins. Yet, while no new epitope emerged within loop 150, this region represents an epitope present in both pandemic and non-pandemic proteins, while its conformation changed nonadaptively in the 2009 sequences (swine and humans).

On the other hand, the comparison of predicted B-cell epitopes of swine sequences *vs*. human pandemic sequences (Figure 5B) shows that the predicted epitopes are almost identical between these two viruses, to the exception of a small region N-terminal region 37-43. Importantly, none of the sites potentially under positive selection falls within a predicted epitope difference. As a consequence, the emergence of novel NA epitopes in the 2009 H1N1 pandemic viruses is most likely nonadaptive.

## Conclusions

To summarize the results found in this study, we showed that (i) the only evidence for positive selection is in the HA and NA antigenes during host-switch events to human hosts, while the emergence of the pandemic was nonadaptive (to the virus), (ii) loop 150, which contains the active site of NA, is an epitope present in all sampled sequences (swine, human non-pandemic and human pandemic viruses), and a recent substitution in this epitope (V149I) spread rapidly but nonadaptively through the viral NA sequences in 2009 irrespective of their host, potentially by means of a demographic bottleneck in human viruses following a reassortment event with a virus of swine origin [11], (iii) this substitution (V149I) caused a structural modification of the 2009 pandemic NA protein, and (iv) although this loop 150 is found in all viruses sampled here, the pandemic NA proteins are predicted to possess four novel epitopes not found in viruses circulating in swines or non-pandemic humans.

These results are significant on two fronts. First, in terms of methodology, the use of codon models proved here to return more sensible results than the use of population genetics test of neutrality such as the MKT or the Tajima test, which both have limitations when it comes to analyzing viral data as population genetics-based tests can be sensitive to demographic changes and/or high levels of diversity, so that these tests can have low power [23,34]. However, the use of codon models in the context of a population study is also not fully satisfactory, as not all nonsynonymous substitutions can be assumed to be fixed. To circumvent this limitation, mutation-selection models that aim at bridging the gap between these two evolutionary scales have been developed [35-37]. However, mutation-selection models have, so far, not been extended to detecting selection at certain amino acid sites in particular lineages and thus, do not allow us to investigate the complex question as to when (the lineages) and where (the amino acid sites) evidence for positive selection can be found.

Second, in terms of the biology of Influenza A viruses, our study shows that, while host-switch events and pandemics are not tightly coupled, it is likely that the emergence of new epitopes in a population is first nonadaptive for the virus. Then, the frequency of these epitopes increases very quickly due to drift, before plummeting again because they are linked to a highly virulent phenotype that kills its hosts too quickly. A similar argument is generally derived from modeling the population dynamics of Influenza viruses, potentially including very sophisticated immune interactions between hosts and viruses (e.g., [38]), and was also put forward based on the study of H3N2 subtypes [39]. It is also significant to note that, while certain amino acid substitutions may be linked to the emergence of a particular epidemic or pandemic Influenza strain, such as V149I, the persistence or re-emergence of this very same substitution is no guarantee of an up-coming threat to public health. More likely, the emergence of an epidemic or pandemic 'phenotype' is the result of epistatic interactions between sites within [17] or across segments, forming a constellation or network of epistatic interactions that are changing over time in ways that we do not currently fully understand. It is tempting to associate these changes of epistatic interactions to antigenic shifts, and future studies should address this potential link. The observation that evidence for positive selection was found only in the HA and NA genes, which are two of the genes for which *N_e_τ *increases, while no evidence for positive selection was found in the other genes showing such an increase may suggest that HA, NA, PB1 and the M and NS genes might be linked. Yet, this linkage is not constant in space or in time as these five different segments in 2009 H1N1 viruses have different origins, with HA and NS coming from classical swine, while NA and M come from an Eurasian avian-like swine and PB1 comes from a human H3N2 virus [2]. A better insight into the timing and the forces at play in the emergence of Influenza viruses and into the dynamics of gene constellations in Influenza viruses will require a continuous and in-depth surveillance of the viruses circulating around the world in its various hosts [11].

## Methods

### Data collection

Complete Influenza A H1N1 genomes were downloaded from the National Center for Biotechnology Information based on the genomeset file ftp://ftp.ncbi.nih.gov/genomes/INFLUENZA/ in February 2010. The extracted genomes were sampled subject to the following constraints: (i) collected between 2000 and 2010, inclusively, (ii) from Mexico, the USA and Canada, and (iii) from human and swine hosts. This resulted in 1180 complete genomes (with no genome from 2010), for which we extracted the ten standard or 'canonical' protein-coding sequences (PB2, PB1, PA, HA, NP, NA, M2, M1, NS2 and NS1). Each of them was aligned with Muscle [40] based on their amino acid translations [41]. Only complete sequences were considered at this stage. Manual adjustments were performed, in particular for genes on the last two segments (M2, M1, NS2 and NS1) and the second segment, for which some sequences are not properly annotated; misaligned sequences were removed. The final alignments contained 1142, 1172, 1170, 1164, 1173, 1158, 1154, 1154, 1163 and 1163 sequences, respectively for PB2, PB1, PA, HA, NP, NA, M2, M1, NS2 and NS1. Accession numbers are listed in Additional file 3.

### Clustering of sequences

Because this large number of sequences would be problematic for phylogenetic analyses, we reduced the size of these alignments by clustering sequences by similarity. Two steps were involved. First, we constructed a matrix of pairwise distances with PAUP [42] under the GTR + Γ + I model of evolution for each alignment. Sequences in the resulting matrices were then clustered with DOTUR [43] using the nearest neighbor algorithm. Sequences similar at the 0.01% level were then discarded, which resulted in alignments containing 53, 57, 54, 78, 54, 76, 111, 70, 142 and 90 sequences, respectively for PB2, PB1, PA, HA, NP, NA, M2, M1, NS2 and NS1. Alignments are available at http://www.bioinformatics.uottawa.ca/stephane.

### Phylogenetic reconstruction and detection of host-switch events

For each alignment, we selected the appropriate model of evolution with the Akaike Information Criterion [14]. Because we need rooted trees to map ancestral host-switch events, we used "relaxed molecular clocks" as implemented in BEAST [12] to estimate the rooted phylogeny of each of the ten genes. The priors were set as follows for all ten analyses. The uncorrelated lognormal model of rate change was used [44], and mean rates were estimated. A coalescent Bayesian skyline model with ten breakpoints and linear splines was used as a prior for speciation times [45]. Substitution models with a "+Γ" component used a discrete gamma distribution with four rate categories. The Markov chain Monte Carlo samplers were run for 100 million steps with a thinning of 2500 steps, except for PB1 for which samplers were run for 500 million steps to circumvent convergence issues. Each sampler was run in duplicate to check for convergence. Burn-in periods were determined graphically with Tracer http://tree.bio.ed.ac.uk/software, set conservatively to 10 million (100 million for PB1), discarded from the log files which were then combined across the two replicates for each gene and used to produce the ten consensus gene trees, rooted by construction.

These trees were then used to map host-switch events, reconstructing manually the most parsimonious mappings. Given that only a small number of events were present on the tree, this procedure is unlikely to underestimate the number of switches. In what follows, only host-switch events from swine to human were marked in the tree files, since we are only interested in detecting positive selection related to that particular switch. The sporadic host-switch events from human to swine were left unmarked.

### Tests of positive selection and of neutrality

The test of positive selection described in [46] was used to detect site potentially under positive selection in the branches on which host-switch events were located. Briefly, nonsynonymous to synonymous rate ratios, denoted *ω*, are used to measure selection in protein-coding genes, with *ω *< 1 indicating negative selection, *ω *= 1 neutral evolution and *ω *> 1 positive selection [20]. A branch-site codon model allows the *ω *rate ratio to vary along the sequence in some pre-specified branches, called the foreground branches, while the ratio in the other branches, or background branches, is kept constant and < 1 [47]. The likelihood ratio test (LRT) used here compares a null model that does not allow for positive selection in the foreground branches to a model that allows positive selection at some sites in the foreground branches [46]. To be conservative, the LRT test statistic was assumed to follow a χ ^2 ^distribution with one degree of freedom rather than the appropriate mixture distribution [46]. Sites potentially evolving adaptively were inferred with a Bayes empirical Bayes method [29] at the 95% posterior probability cutoff. These analyses were performed for each of the ten genes. All analyses were run, with PAML ver. 4.2b [48], in duplicate starting from random initial values, in order to check for convergence.

Neutrality was first tested with the McDonald-Kreitman test as implemented online at http://bioinf3.uab.cat/mkt/mkt.asp[49]. The alignments were used as computed above, and divergences were corrected with the Jukes and Cantor model, which is similar in spirit to a recently proposed method [23]. The *p*-values were computed as a χ ^2 ^homogeneity test on a contingency table. The Tajima test [50] was performed with the R package pegas [51]. Only *p*-values based on the normal distribution are reported. The neutrality tests for the human 2009 H1N1 pandemic were run on sequences from the pandemic clade (all the remaining sequences were used as outgroup sequences for the MKT), while the test for host-switch events was run on human sequences (swine sequences were used as outgroup sequences for the MKT).

### Epitope and structural predictions

In order to identify linear B-cell epitopes, that is, contiguous amino acids in an antigen (NA here) that are recognized by the antibodies of the human (host) immune system, we used the BepiPred online server http://www.cbs.dtu.dk/services/BepiPred[52]. This machine learning method is based on the combination of a hidden Markov model with a propensity scale method, and was originally trained on three independent data sets. For each amino acid position in an alignment, a prediction score is calculated [52], and site assignment to a linear B-cell epitope is made where the score is above a certain threshold. Different thresholds give different sensitivities (*Sn*) and specificities (*Sp*); we used the default threshold of 0.35 that corresponds to *Sn *= 0.49 and *Sp *= 0.75 [52]. The NA pandemic sequences were translated from CY052236, CY044074, GQ465697, CY054789, CY052729, CY052593, GQ402235, CY052705, CY050881, CY053073, CY053263, CY050330, GQ465702, CY040890 and CY052809, while the non-pandemic sequences were obtained from DQ889687, GQ200251, CY026221, CY025941, CY025391, CY026893, CY025215, CY027045, CY028061, CY026877, CY028325, CY050478, CY038772 and CY026373. The swine sequences had accession numbers AY619960, DQ280202, DQ280218, DQ280194, DQ280251, DQ280242, GQ150330, CY053647, GQ484356, FJ611900 and EU604690. For each of these three sets of sequences, the score at each site was averaged over the sequences.

Tridimensional (3D) structures were predicted with Swiss-Model [31], using the translation of CY052236 for the pandemic target sequence, DQ889687 for the non pandemic target, and DQ280194 for the swine target. Root mean square deviations (RMSDs) were calculated with SPDBviewer [53] based on C*^α ^*atoms, and 3D models were plotted with KiNG available at http://kinemage.biochem.duke.edu. Structural models are available in Additional file 4.

## Authors' contributions

SAB conceived of the study, JA and SAB performed the research, JA and SAB wrote the manuscript. All authors read and approved the final manuscript. 

## Supplementary Material

Additional file 1 **The ten reconstructed phylogenetic trees, with branch lengths in units of time (years before 2009)**. Click here for file

Additional file 2 **The ten reconstructed phylogenetic trees, with branch lengths in units of expected numbers of substitutions per nucleotide site**. Click here for file

Additional file 3 **List of the accession numbers of the sequences from the 1180 genomes used in this study**. Click here for file

Additional file 4 **PDB files (zipped) containing the three-dimensional models of the protein structures of NA swine (**SwineModel.pdb**), non-pandemic (**NonPandemicModel.pdb**) and pandemic human (**PandemicModel.pdb**)**. Click here for file
