# Supplementary figures and images for: The nonadaptive nature of the H1N1 2009 Swine Flu pandemic contrasts with the adaptive facilitation of transmission to a new host
Source: BMC Evol Biol. 2011 Jan 6;11:6. doi: 10.1186/1471-2148-11-6 (PMC3024937; doi:10.1186/1471-2148-11-6)

PB2

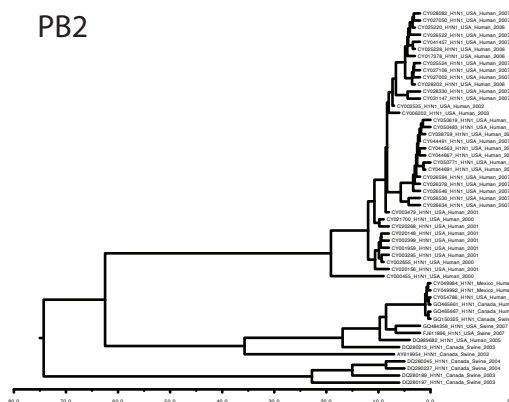

PB1

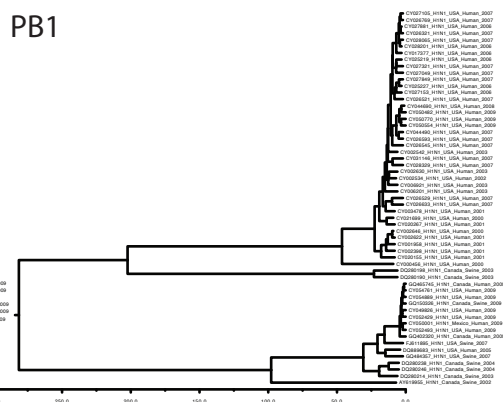

PA

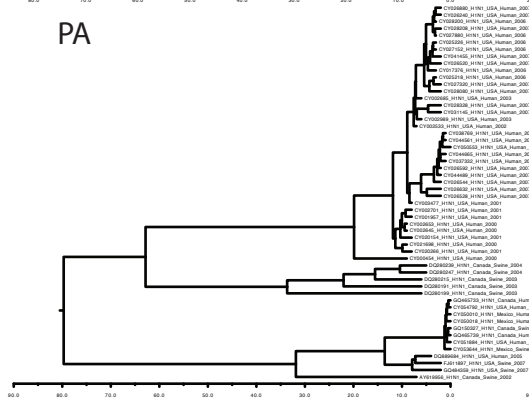

HA

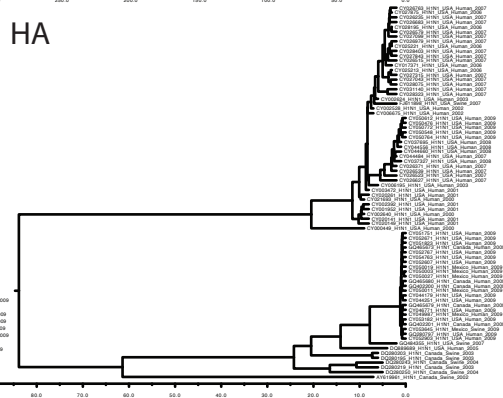

NP

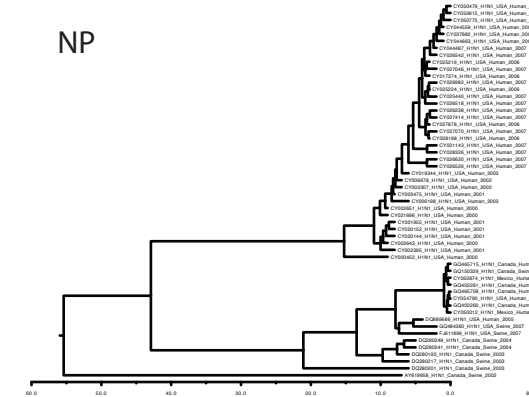

NA

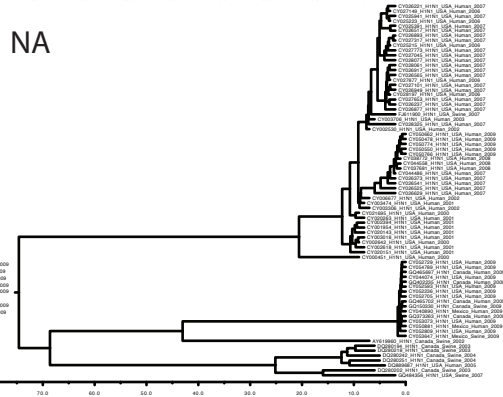

M2

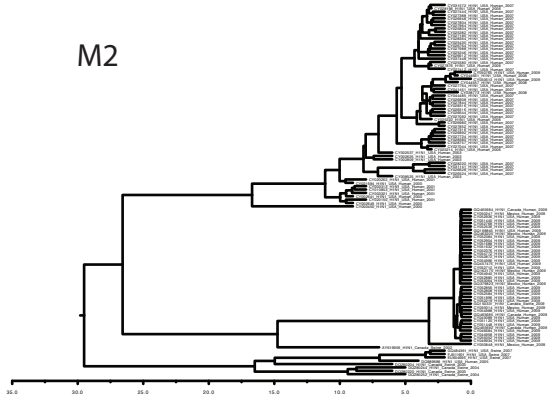

M1

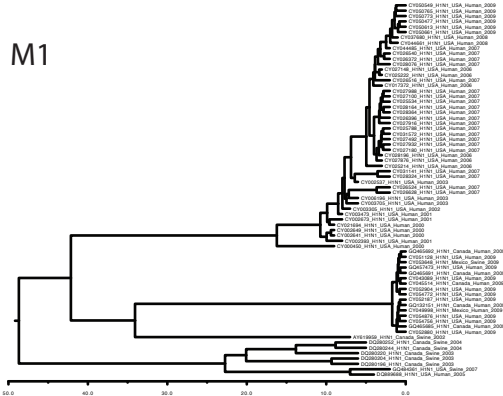

NS2

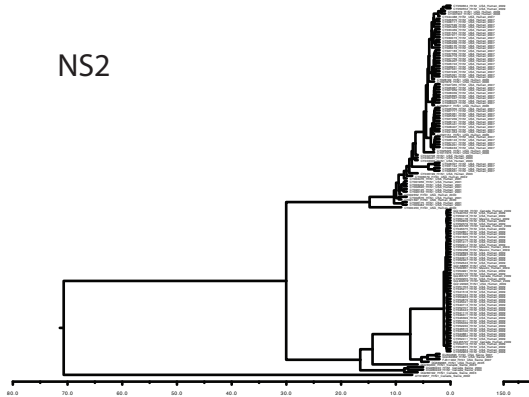

NS1

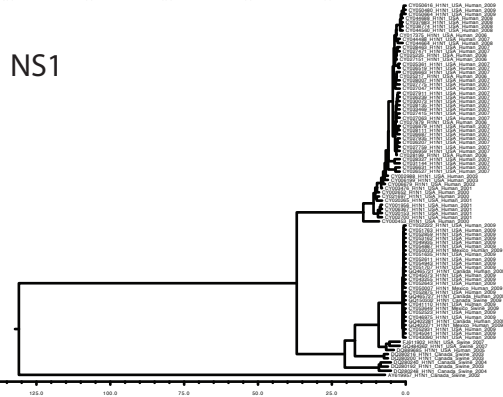

Supplement: Additional file 1 — The ten reconstructed phylogenetic trees, with branch lengths in units of time (years before 2009). [file 1471-2148-11-6-S1.PDF]

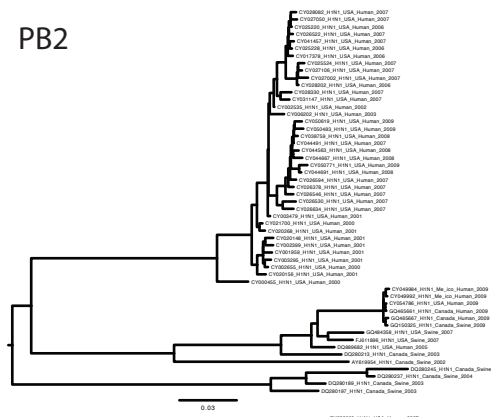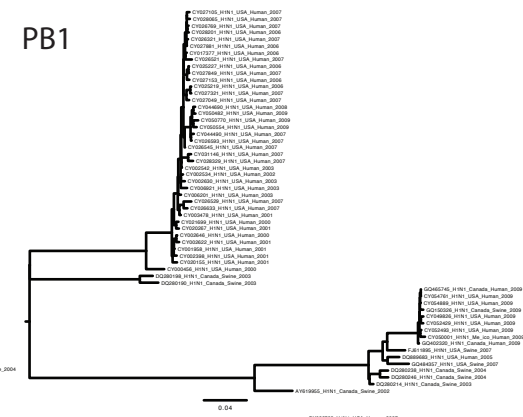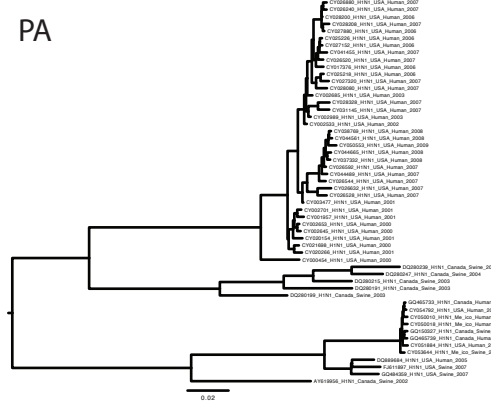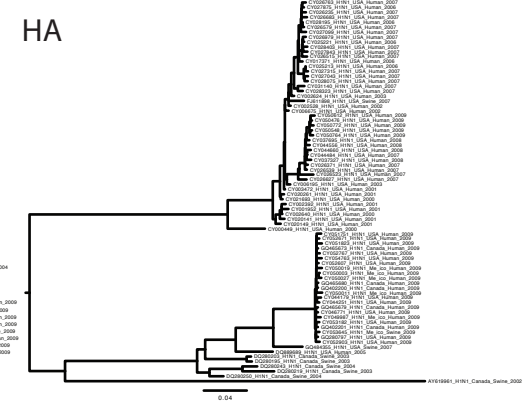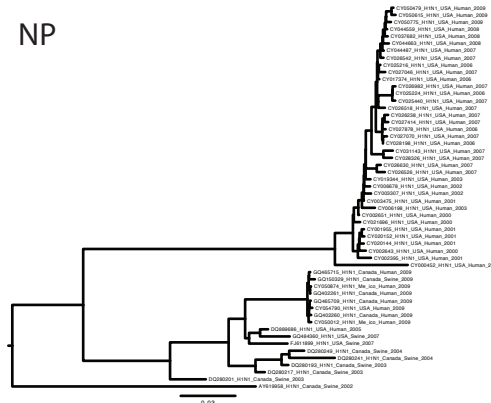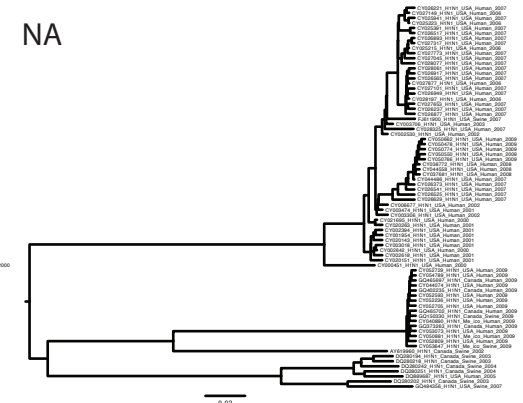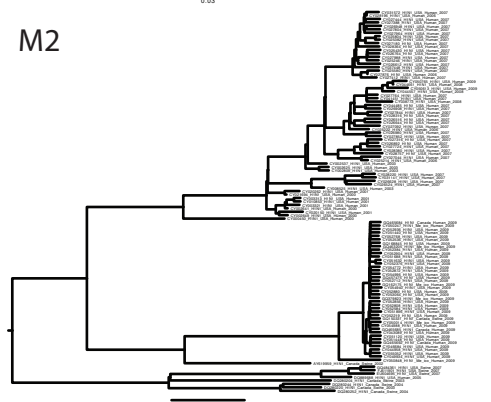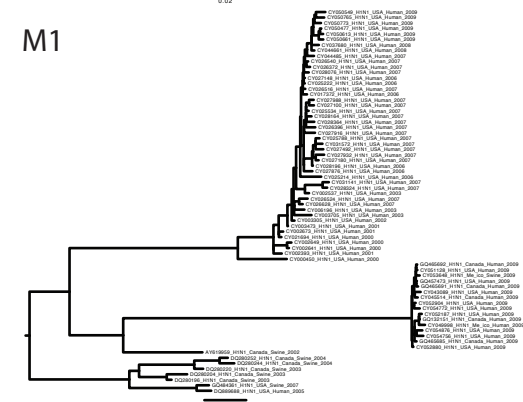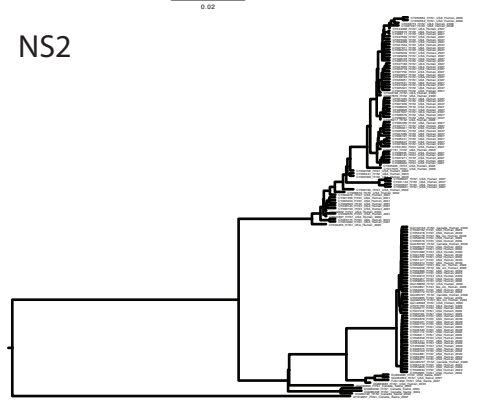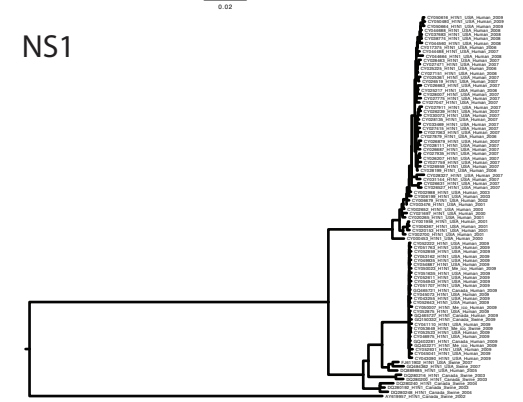

Supplement: Additional file 2 — The ten reconstructed phylogenetic trees, with branch lengths in units of expected numbers of substitutions per nucleotide site. [file 1471-2148-11-6-S2.PDF]
